# For Grandparents’ Sake: the Relationship between Grandparenting Involvement and Psychological Well-Being

**DOI:** 10.1007/s12126-017-9320-8

**Published:** 2018-01-16

**Authors:** Eon-Ha Park

**Affiliations:** Department of Social Welfare and Early Child Education, Daedong College, Geum-Jeong-Gu, Dong-Bu-Gok-Ro 27, Beon-Gil 88, Busan, 46270 South Korea

**Keywords:** Grandparenting, Role-meaning, Level of involvement, Burden of care, Psychological well-being

## Abstract

The study examined the impact of role type and involvement level on psychological wellbeing among 255 South Korean grandparents. Participants in non-baseline role types (those who participated in grandparenting) tended to perceive meaning in their lives and to exhibit relatively low levels of stress and depressive mood. With respect to involvement, stress tended to decrease (β = −0.134) when this variable increased, but no relationship was found with perceived meaning or depressive mood. In addition, the hypotheses that burden of caring for grandchildren would mediate the impact of role type and involvement level on psychological wellbeing, and that respect from adult children would moderate this mediation, were supported. Four policy and practice implications are identified. First, policy makers should provide resources for seniors so that those who might benefit from actively nurturing their grandchildren can do so more readily. Second, given that significant moderated mediation emerged in terms of care burden and grandparent roles and involvement, practitioners should be aware of the interactions among grandparents, children, and grandchildren when providing counselling and other resources. Third, the study suggests the importance of applying dynamic practice models, particularly in a context like South Korea, where most families encompass more than two generations. Finally, the results have implications regarding the impact of grandparent attitudes and behaviour patterns within changing social dynamics, and practitioners should be prepared to assist clients and their families to address their evolving roles and the impacts they have on the family unit. Limitations and implications for future research are also discussed.

## Introduction

While societal changes may increasingly undermine roles traditionally played by grandparents, improvements in healthcare, hygiene, and nutrition have substantially increased average life expectancies. As a result, many grandparents are living longer and remaining healthy and active into old age. Additionally, changes in working conditions associated with urbanization, along with the employment of women outside the home, have forced many families to depend more heavily on grandparents for primary caregiving of their grandchildren while the parents are at work (Cherlin and Furstenberg 1986). In South Korea, where the present study was conducted, a considerable rise in suicides among economically challenged elderly individuals occurred over two decades in the late twentieth and early twenty-first century (Seoul Statistical Office 2005). In this light, studies that inquire into the potential impact of grandparenting roles on health and happiness can contribute to the understanding of whether certain types or levels of grandparenting involvement should be considered as potential risk factors—or, perhaps, as potential factors mitigating against risk—for elderly individuals.

To contribute to the understanding of how caregiving affects grandparents, the study examined the impact of four previously identified grandparenting role types, along with involvement level, on grandparents’ psychological well-being. Psychological well-being was measured through depressive mood and stress indices, as well as the extent to which participants perceived meaning in their lives. Hypotheses reflected the expectation, based on a review of relevant literature, that childcare burden would act as a mediator on the relationship between grandparenting role type/involvement level and psychological well-being. Moreover, the study also found that respect accorded to grandparents (by the parents) could moderate this mediating effect of childcare burden.

### Types and Impact of Grandparenting Involvement

The present study was designed to potentially capture both positive and negative impacts of a variety of forms and levels of caregiving on grandparents’ psychological well-being. Notably, many previous studies assessing the grandparent-grandchild relationship have focused on the adverse consequences of custodial grandparenting. Contributing factors identified in this regard include inadequate living conditions experienced by custodial grandparents, difficulties experienced when caring for grandchildren, and problematic behaviours among grandchildren raised by custodial grandparents (Kwon 2000; Ohk 2005). Negative health effects of stress and depression experienced by grandparents caring for their grandchildren are also discussed by Burton (1992), Goodman and Silverstein (2002), Kelley et al. (2000), Minkler et al. (1997), Rodgers-Farmer (1999), Strawbridge et al. (1997), and Szinovacz et al. (1999).

Indeed, grandparents who participate substantially in caring for their grandchildren often face stressors that can adversely affect their health and well-being. Moreover, in addition to childrearing pressures, grandparents who anticipated a leisurely retirement—or expected to play more companionate roles with their adult children—may resent the demands associated with being expected to raise their grandchildren. In this regard, many grandparents who take on a major childcare role complain of feeling ‘off track’ (Neugarten 1976), which is to say that they perceive their current caregiver role as inconsistent with their late life expectations. Hence, they may feel physically unable to cope with raising young children (Goodman and Silverstein 2002). Similarly, pressures can arise from the fact that grandparents may lack knowledge or experience regarding new technologies with which their grandchildren are (or need to be) familiar. In other words, grandparents may not recognize or understand the skills that their grandchildren will need in order to succeed in the modern workplace (Strom et al. 2008).

Although grandparents face many challenges and incur social and personal costs when raising their grandchildren, such relationships can also entail benefits for the caregiver. Indeed, researchers have long been aware that many grandparents feel psychologically rewarded through helping their adult children and that they appreciate the opportunity to redress their perceived failures with their own children (Neugarten et al. 1968). According to a number of more recent studies, moreover, the rewards of raising grandchildren can include feeling younger, perceived or actual increases in longevity, finding meaning in life, reforming familial and other relationships, and achieving personal satisfaction, intimacy, and love (Chen and Silverstein 2000; Cox 2000; Jendrek 1994).

Gonzalez and Anuncibay (2008) looked at grandparents’ role as caregivers in the context of a variety of tasks that grandparents might engage in with their grandchildren. In a survey of 603 grandparents in Burgos, Spain, the researchers found that while many grandparents were engaged in looking after their grandchildren, they also commonly engaged in such leisure activities as reading together, playing, or going to the park. The authors also noted that grandparents often assumed the role of caregiver in response to the parents’ needs rather than their own needs as grandparents, and that most grandparents saw their primary role as grandparents as consisting of one or more of the following: a) playing with their grandchildren, b) indulging them, c) providing them with unconditional love.

While nurturing their grandchildren, grandparents sometimes find that their lives become more fulfilling as they find new meaning and purpose in life. In other words, while sharing their wisdom and experience with their grandchildren, they often find meaning in their own existence, and their sense of pride increases as they perceive themselves as people who play essential roles in their grandchildren’s growth and development (Kennedy and Keeney 1988; Kim 2006). They perceive grandparenting as an opportunity to experience, though perhaps differently, some of the diverse emotions that they experienced in the past, and they may feel a sense of satisfaction at the opportunity to nurture their grandchildren in ways that they may not have been able to nurture their children (Burton 1992). Thus, grandparents sometimes consider even their grandchildren’s mischievous acts or lack of respect as a consolation in life (Lee 2005).

Indeed, studies have shown that most grandparents express that they find new meaning in life when nurturing their grandchildren, but it is also known that many have mixed feelings about their role in acting as a proxy for the grandchildren’s parents (Kornhaber and Forsyth 1994). Grandmothers, in particular, experience more negative feeling compared to grandfathers; moreover, although grandparents are typically grateful for the opportunity to nurture their grandchildren and value this role, Minkler et al. (1997) also found that most grandparents not only manifested emotional conflict but also experienced depression and burnout. Nonetheless, these authors found that grandparents’ nurturing of their grandchildren could enrich the grandparents’ lives by giving them new meaning, and that close interaction with their grandchildren could have a positive impact on the grandparents’ emotional wellbeing.

### Hypotheses

According to social exchange theory, grandparenting takes place within the context of an intergenerational balance (Marbach 1995). For instance, grandparents provide support and care for their grandchildren (e.g., babysitting and education) in exchange for various forms of assistance (e.g., instrumental and emotional support) from their adult children. However, social exchange theory is based on an economic model of human behaviour, and critics have argued that this theory does not account for social norms or emotions that promote a sense of familial solidarity, or even altruistic behaviour (Park 2012). In the Korean context, moreover, it is important to take account of Confucian moral values, according to which the family is both the core of and the model for social organization (Fan 2006). Thus, each individual bears a responsibility for maintaining familial integrity, and this responsibility does not depend on—and cannot be reduced to—a balance between individual interests.

**Fig. 1:**
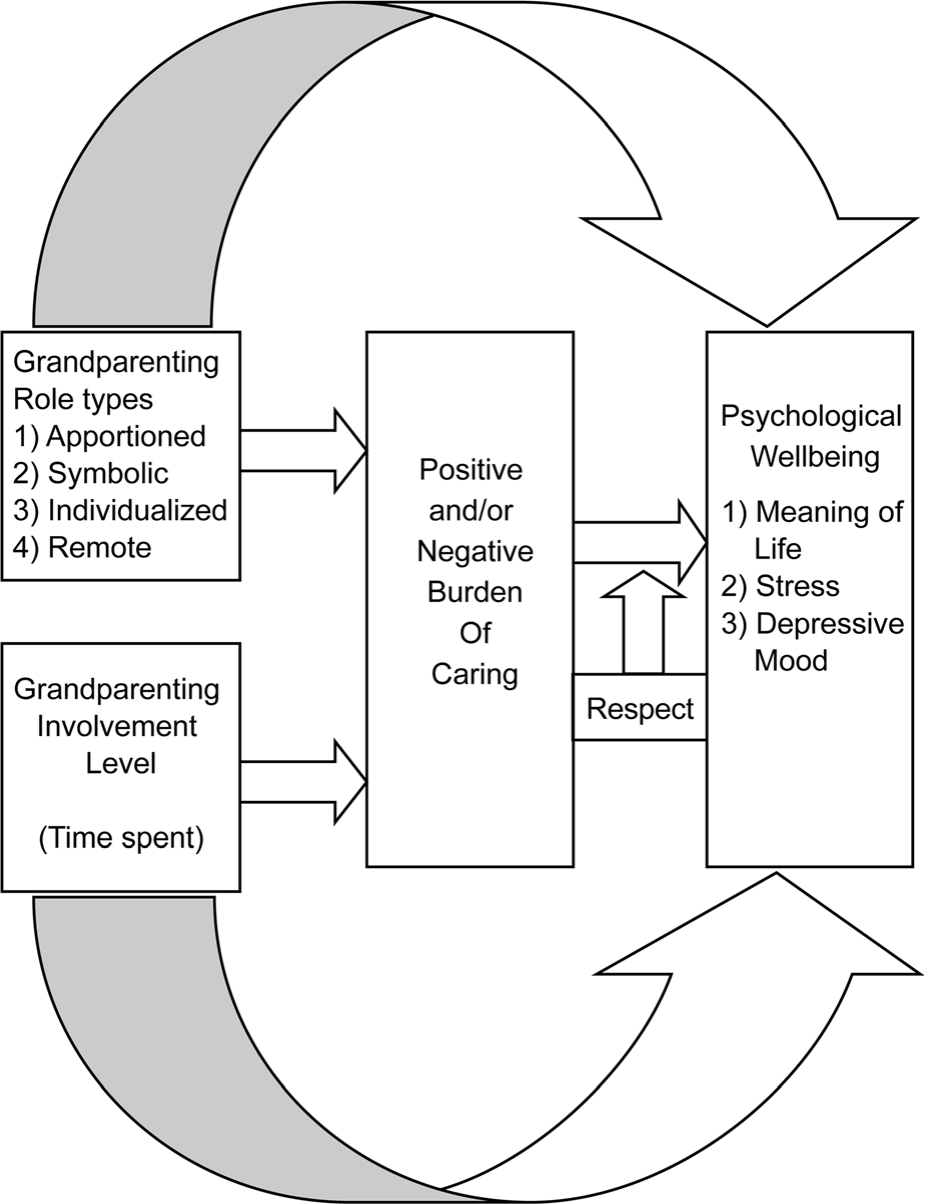
Impact of role type, involvement level, burden, and respect on grandparents’ psychological well-being

In framing the study’s hypotheses, it was therefore understood that grandparents provided nurturance to their grandchild—actively or otherwise—for the same reasons that a parent nurtures the child, as well as that the adult children (typically) demonstrated deference and respect toward the grandparents. Figure 1 illustrates the relationship among the key variables in the study, i.e., grandparenting role type, involvement level, and psychological well-being, as well as the relationships among these variables, the mediating factor of childcare burden, and the moderating factor of respect accorded to grandparents (by the parents). Thereafter, the hypotheses are stated and explained.

- *Hypothesis 1*. Grandparent role types and involvement level will have a relationship to their psychological wellbeing.

$$ {\displaystyle \begin{array}{l}\mathrm{Y}1={\mathrm{B}}^0+{\mathrm{B}}^1{\mathrm{X}}^1+\mathrm{Controls}.\\ {}\mathrm{Y}1={\mathrm{B}}^0+{\mathrm{B}}^2{\mathrm{X}}^2+\mathrm{Controls}.\end{array}} $$

- *Hypothesis 2*. Grandparent role types and involvement level will have a relationship to their burden of caring for grandchildren.

$$ {\displaystyle \begin{array}{l}\mathrm{Y}2={\mathrm{B}}^0+{\mathrm{B}}^1{\mathrm{X}}^1+\mathrm{Controls}.\\ {}\mathrm{Y}2={\mathrm{B}}^0+{\mathrm{B}}^2{\mathrm{X}}^2+\mathrm{Controls}.\end{array}} $$

- *Hypothesis 3*. The burden of caring for grandchildren will act as a mediator between the role types and level and psychological wellbeing.

$$ {\displaystyle \begin{array}{l}\mathrm{Y}2={\mathrm{B}}^0+{\mathrm{B}}^1{\mathrm{Y}}^1+{\mathrm{B}}^2{\mathrm{X}}^1+\mathrm{Controls}.\\ {}\mathrm{Y}2={\mathrm{B}}^0+{\mathrm{B}}^1{\mathrm{Y}}^1+{\mathrm{B}}^3{\mathrm{X}}^2+\mathrm{Controls}\end{array}} $$

(Here B^2^ and B^3^ should be close to zero or at least reduce.)

- *Hypothesis 4*. The respect accorded to grandparents will act as a moderator between the burden of caring and psychological wellbeing.

$$ {\displaystyle \begin{array}{l}\mathrm{Y}2={\mathrm{B}}^0+{\mathrm{B}}^1\mathrm{Y}1+{\mathrm{B}}^2\mathrm{X}1+{\mathrm{B}}^3\mathrm{X}2+{\mathrm{B}}^4\mathrm{Z}+{\mathrm{B}}^5\left(\mathrm{Y}{1}^{\ast}\mathrm{Z}\right)+\mathrm{Controls}.\\ {}\mathrm{Y}2={\mathrm{B}}^0+{\mathrm{B}}^1\mathrm{Y}1+{\mathrm{B}}^2\mathrm{X}2+{\mathrm{B}}^4\mathrm{Z}+{\mathrm{B}}^5\left(\mathrm{Y}{1}^{\ast}\mathrm{Z}\right)+\mathrm{Controls}.\end{array}} $$

(B^5^ is significant and large.)^1^

## Methods

### Sample and Recruitment Procedures

To identify prospective participants, 14 kindergartens were selected randomly from a list posted by the Alliance of Kindergartens in Seoul, South Korea (2010). At the request of the researcher, staff at these kindergartens facilitated contact with the children’s grandparents. This study was approved by the ethical review board of the researcher’s home institution. Informed consent was obtained from all individual participants included in the study. Of these, 300 grandparents completed the study questionnaire, which was delivered to the kindergartens by a personal visit or through the mail between May 23 and June 7, 2011. However, data from 45 grandparents had to be excluded because too many questions were omitted or answered arbitrarily, leaving a sample of 255 participants whose data were considered in the study.

### Variables, Measurement, and Operationalization

The main independent variables were *grandparenting role type* and *involvement level*, and the dependent variable was *psychological well-being*. The study also assessed care *burden* (stress) as a mediating variable and *respect* accorded to grandparents as a moderating variable. Involvement was operationalized as the amount of time spent participating in care for their grandchildren, measured based on participants’ responses to the survey question *How much time do you spend with your grandchildren each day?* Psychological well-being was operationalized to include participants’ level of depressive symptoms, satisfaction with life, and perceptions of meaning in life. This variable was measured using the Korean form of the Geriatric Depression Scale (KGDS; Chung et al. 1998), the Family Inventory of Life Events and Changes (FILE) measure (McCubbin et al. 1979), and the Satisfaction with Life Scale (SWLS; Diener et al. 1985). Burden was measured using the Grandparent Caregiver Burden Scale (Pruchno 1999), while items from the Familism Scale (Heller 1970) were used to measure respect.

Grandparent role-meaning was analysed according to the typology of Robertson (1977), who identified and defined four categories, as follows:

- Apportioned type: *The grandparent recognizes a high level of social normative meaning in their role and engages in close interaction with grandchildren.*
- Symbolic type: *The grandparent is well aware of the social normative meaning of their role but does not feel a personal attachment to his/her grandchildren and does not intervene from a behavioural aspect.*
- Individualized type: *The grandparent finds personal meaning, even when the grandparenting role does not carry normative meaning.*
- Remote type: *The grandparent does not carry out the role of a ‘grandparent’, even from a behavioural aspect, since this type involves almost never recognizing the social normative meaning of being a grandparent*.

To determine participants’ role type, a 12-item Likert-scale measure was used that included factor statements for each of two dimensions: personal and social. The personal dimension values personal normative meaning, whereas the social dimension values social normative meaning (see Table 1) (sample factor statements for both dimensions are provided under the Results section). Grandparents who scored high on both the personal and social dimensions were assigned to the *apportioned* type. Those with low scores on both dimensions represented the *remote* type. Those who scored high on the personal but low on the social dimension were assigned to the *individualized* type. Those who scored low on the personal but high on the social dimension were assigned to the *symbolic* type. Finally, a factor analysis was conducted to ascertain the independence of these two dimensions based on item clustering.

**Table 1:** Grandparent Role-meaning (Robertson 1977)

		Personal	
		High	Low
Social	High	Apportioned	Symbolic
	Low	Individualized	Remote

### Data Analysis

Chi-square (χ^2^) tests were conducted to examine the distribution of the four grandparenting role types in terms of general participant characteristics. A principal component analysis was used for the factor analysis, with factor loading cut-offs of 0.55, and a varimax rotation was used. A hierarchical regression analysis was conducted to verify the mediating effect of care burden and to measure the impact of grandparenting role type and involvement level on psychological well-being. The demographic variables (participant characteristics: gender, age, spouse, religion, education, number of children, housemates, material support, health, and living standard) were controlled. Based on Baron and Kenny (1986), mediation effects were assessed using a Sobel test.

## Results

### Sample Characteristics

Table 2 summarizes general demographic characteristics of the sample. Of the 255 participants, 87.8% were women, and 12.2% were men. The age group between 61 and 65 years had the highest number of participants (37.6%), followed by those aged between 66 and 70 years old (36.5%) and over the age of 70 (16.1%), respectively. Of the participants, 81.6% were married. The largest group of participants identified themselves as Catholic (47.1%), followed by Protestant (25.5%), Other (14.1%), and Buddhist (13.3%). Regarding education, the majority had a high school diploma (48.6%), followed by those with a university diploma (33.3%) and those who did not complete high school (18.0%). Grandparents with four or more grandchildren represented 43.5% of the sample, while 37.3% had three and 19.2% had two grandchildren or fewer. Participants who lived with a spouse accounted for 66.7%; 22.0% lived with others, and 11.4% lived alone. In terms of income, the largest group (52%) lived on ‘Property’ income (savings, rent, etc.), followed by ‘Working’ (24.7%), support from children (9.4%), their own pensions or severance pay (9%), and ‘Other’ (4.7%). Participants who self-reported as being ‘Healthy’ accounted for 57.3% of the sample, versus ‘Normal’ (38.8%) and ‘Unhealthy’ (3.9%). Finally, with respect to living standard, 60.4% considered themselves as having a mid-level living standard, while 36.5% claimed a high-level living standard, and 3.1% considered themselves as having a low-level standard of living.

**Table 2:** Participant characteristics

Category		Frequency (Number of participants)	Percent of sample (%)
Gender	Male	31	12.2
	Female	224	87.8
Age	Less than 60 years	25	9.8
	Between 61 and 65 years	96	37.6
	Between 66 and 70 years	93	36.5
	More than 70 years	41	16.1
Spouse	Yes	208	81.6
	No	47	18.4
Religion	Buddhist	34	13.3
	Protestant	65	25.5
	Catholic	120	47.1
	Other	36	14.1
Education	Less than high school education	46	18.0
	High school education	124	48.6
	University education	85	33.3
Number of children	2 or fewer	49	19.2
	3	95	37.3
	4 or more	111	43.5
Housemates	Live alone	29	11.4
	Live with a spouse	170	66.7
	Live with others	56	22.0
Material support	Working	63	24.7
	Property income (savings, rent etc.)	133	52.2
	Pension or severance pay	23	9.0
	Supported by children	24	9.4
	Other	12	4.7
Health	Healthy	146	57.3
	Normal	99	38.8
	Unhealthy	10	3.9
Living standard	High	93	36.5
	Middle	154	60.4
	Low	8	3.1
Total		255	100.0

As a baseline for comparison, according to the 2011 census (Seoul Statistical Office 2011), 11% of the population of Seoul, South Korea was over the age of 65, and 43% of these individuals lived with a spouse or alone. Approximately 54.2% reported having more than a high school education, while 52.7% provided their own material support. Thus, although comparators are not available for all categories, the present sample characteristics appear to match those of the overall South Korean population fairly well.

### Chi-Square Analysis

The distribution of the four grandparenting role types across the study sample was found to be as follows: apportioned type (*n* = 125), remote type (*n* = 100), symbolic type (*n* = 23), and individualized type (*n* = 8). Chi-square analyses were performed to examine the distribution of these types in terms of the participant demographics. The results are shown in Table 3.

**Table 3:** Distribution of the four role types by participant characteristics

		ROLE TYPE (no. (%))						
Category		Apportioned	Symbolic	Individualized	Remote	Total	χ^2^ (df)	*p*
Gender	Male	2(6.5)	0(0)	1 (3.2)	28 (90.3)	31(100)	39.598 (3)	.000
	Female	122(54.5)	23(10.3)	7 (3.1)	72 (32.1)	224(100)		
Age	60 or younger	1(4.6)	4(16.0)	4(16.0)	16 (64.0)	25(100)	58.586 (9)	.000
	61 to 65	54(56.3)	1(1.0)	3 (3.1)	38 (39.6)	96(100)		
	66 to 70	49(52.7)	18(19.4)	0 (.00)	26 (28.0)	93(100)		
	71 or older	64(48.8)	0(0.0)	1(2.4)	20 (48.8)	41(100)		
Spouse	Yes	103 (49.5)	23 (11.1)	6(2.9)	76 (36.5)	208(100)	7.674 (3)	.053
	No	21 (44.7)	0 (0.0)	2 (4.3)	24 (51.1)	47(100)		
Religion	Buddhist	1 (2.9)	1(2.9)	2 (59)	30 (88.2)	34(100)	132.411 (9)	.000
	Protestant	41(63.1)	0 (0.0)	4 (6.2)	20 (30.8)	65(100)		
	Catholic	80(66.7)	22 (18.3)	1 (0.8)	17 (14.2)	120(100)		
	Other	2 (5.6)	0 (0.0)	1 (2.8)	33 (91.7)	36(100)		
Education	Less than high school education	2 (4.3)	0 (0.0)	2 (4.3)	42 (91.3)	46(100)	102.233 (6)	.000
	High School	81 (65.3)	2 (1.6)	2 (1.6)	39 (31.5)	124(100)		
	University Education	41 (48.2)	21 (24.7)	4 (4.7)	19 (22.4)	85(100)		
Number of Children	2 or fewer	4 (8.2)	1(2.0)	5(10.2)	39 (79.6)	49(100)	76.266 (6)	.000
	3	55 (57.9)	2 (2.1)	2 (2.1)	36 (37.9)	95(100)		
	4 or more	65 (58.6)	20 (18.0)	1 (0.9)	25 (22.5)	111(100)		
Housemates	Live alone	20 (69.0)	0 (0.0)	1 (3.4)	8 (27.6)	29(100)	82.910 (6)	.000
	Live with a spouse	102 (60.0)	21(12.4)	5 (2.9)	42 (24.7)	170(100)		
	Live with others	2 (3.6)	2(3.6)	2 (3.6)	50 (89.3)	56(100)		
Material Support	Working	42 (66.7)	0(0.0)	2 (3.2)	19 (30.2)	63(100)	113.385 (12)	.000
	Property Income	81 (60.9)	22 (16.5)	0 (0.0)	30 (22.6)	133(100)		
	Pension or Severance Pay	0 (0.0)	1(4.3)	2 (8.7)	20 (87.0)	23(100)		
	Supported by children	0 (0.0)	0 (0.0)	2 (8.3)	22 (91.7)	24(100)		
	Other	1 (8.3)	0 (0.0)	2 (16.7)	9 (75.0)	12(100)		
Health Status	Healthy	84(57.5)	21(14.4)	4(2.7)	37 (25.3)	146(100)	41.966 (6)	.000
	Normal	40 (40.4)	2 (2.0)	4 (4.0)	53 (53.5)	99(100)		
	Unhealthy	0 (0)	0 (0)	0 (0)	10 (100)	10(100)		
Standard of living	High	80 (86.0)	1 (1.1)	2 (2.2)	10 (10.8)	93(100)	90.639 (6)	.000
	Medium	44 (28.6)	22 (14.3)	6 (3.9)	82 (53.2)	154(100)		
	Low	0(0.0)	0 (.00)	0 (0.0)	8 (100)	8(100)		

As seen in Table 3, all demographic variables except ‘living with a spouse’ differed significantly depending on role type. The remote grandparenting type was most common among male participants (90.3%), whereas the apportioned type was most frequent for females (54.5%). Relatively few participants were identified in the symbolic and individualized types for both genders.

Participants under the age of 60 were predominately remote-type grandparents (64.0%), while those between the ages of 61 and 65 were predominately apportioned type (56.3%), as were those between the ages of 66 and 70 (52.7%) and aged 71 and above (48.8%).

Those who self-identified as Protestant (63.1%) and Catholic (66.7%) predominately demonstrated an apportioned role; those who self-identified as Buddhist (88.2%) and Other (91.7%) predominately demonstrated remote type. Participants with less than a high school education had higher incidence of remote role (91.3%), while those with a high school (65.3%) and university education (48.2%) predominately reported the apportioned type. Participants with two or fewer grandchildren were more typically remote (79.6%), while those with three (57.9%) and four or more (58.6%) were predominately apportioned. Participants who lived alone (69.0%) or with a spouse (60.0%) were predominately apportioned, while those who lived with others were predominately remote (89.3%). In terms of material support, participants who worked (66.7%) or had property income (60%) were predominately apportioned. Those with pensions or severance pay (75%), those supported by their children (91.7%), and those who answered Other (75%) were predominately remote (χ^2^ = 113.385, *df* = 12. *p* < 0.001). Participants who self-identified as healthy were predominately apportioned (57.5%), while those who self-identified as normal (53.5%) and unhealthy (100%) were predominately remote. Finally, with respect to standard of living, participants with a high standard were predominately apportioned (86%), while those with a medium (53.2%) or low (100%) standard were predominately remote.

### Hierarchical Regression of Mediation and Interaction Effects

In this study, a hierarchical regression analysis was also conducted to verify the mediating effect of burden of caring on the impact of grandparent role type and involvement level on psychological wellbeing, as indicated by the measures specified above (meaning of life, stress and depressive mood). A Sobel test was also conducted to verify the significance of the mediating effect. According to Baron and Kenny (1986), three conditions must be fulfilled in order to establish a mediating effect. In the first equation, the independent variable should affect the mediator. In the second equation, the independent variable should affect the dependent variable. In the third and final equation, the mediator should affect the dependent variable. The process thus involved four steps, which are summarized below.

Step 1: Analysis of the effect on burden of caring of the independent variables: grandparenting role type and involvement level. (burden of caring = B^0^ + B^1^ * independent variable).
Step 2: Analysis of the effect of the independent variables on psychological wellbeing, measured in terms of meaning of life, stress, and depressive mood. (Psychological Wellbeing = B^0^ + B^1^* independent variables).
Step 3: Analysis of the changes in the coefficient value (B) of each independent variable after controlling for burden of caring. (Psychological wellbeing = B^0^ + B^1^ * burden of caring + B^2^ * independent variables).
Step 4: Sobel test to verify the significance of the mediating effect.

### Linearity, Heteroscedasticity, Clustering Correction, Outlier Tests, and Multicollinearity

In order to the check the regression, linearity, heteroscedasticity, clustering correction, outlier tests and multicollinearity diagnostics were used. Searching for outliers was the first check of the diagnostics, and it was performed using a scatterplot of the histogram and a regression standardized residual. Another element of Multiple Regression Analysis is to find the difference between the observed dependent variable and the forecasted dependent variables, which is the residual. The residual should be distributed normally (normality) when it comes to the forecasted dependent variable’s score. At the same time, the residual should assume forecasted dependent variable score and linear correlation (linearity). Also, the residual of the forecasted dependent variable score should be the same for all of the forecasting variables (heteroscadastsicity) (Kennedy 1992). These parameters can be checked using a scatterplot of the forecasted values and residuals to detect extreme values (Kennedy). All of these methods were used in the present study. As a result, data was converted by deviating from normality with respect to the variable *depressive mood*. In order to convert the data, the log was used in this study. With respect to multicollinearity, it is possible to claim that the correlation among independent variables was excessively high. Thus, for this study, the variance inflation factor (VIF) was checked for verification. All of the VIF values were found to be less than 10, making it is possible to claim that there was no multi-collinearity. In the case of clustering correction, hypothesis testing was used for logistic regression in Stata Version 10, with standard errors corrected for clustering within kindergartens. Clustering was used rather than estimating random effects for kindergartens because clustering is more statistically conservative.

### Mediating Effects

First, the association between burden of caring and the independent variables, grandparenting role type and involvement level, was analyzed. All categories of participant characteristics, including gender, age, spouse, religion, education, number of children, housemates, material support, health, and living standard, were controlled.

In this study, the remote grandparenting type was used as a reference group, dummy variable, and baseline against which the three other role types—apportioned, symbolic, and individualized—were compared. This method is reflected in the results presented in the remainder of this chapter.

As seen in Table 4, when the demographic variables (participant characteristics)—gender, age, spouse, religion, education, number of children, housemates, material support, health, and living standard—were controlled, the three other grandparenting role types showed negatively significant compared to the remote type at the significance level of 0.001. The R^2^ was 65.2% and the change of R^2^ was 19.9% for the grandparenting role types. Compared to the remote type, the apportioned type showed the least impact of positive burden of caring (β = −0.766, *p* < 0.001), followed by the symbolic type (β = −0.385, *p* < 0.001) and the individual type (β = −0.148, p < 0.001), respectively.

**Table 4:** Effect of grandparenting role type on burden of caring

	Positive burden of caring			Negative burden of caring		
	B	SE B	β	B	SE B	β
Apportioned type	−1.088	.094	−.766***	−1.206	.137	−.612***
Symbolic type	−.956	.133	−.385***	−.960	.192	−.279***
Individualized type	−.604	.167	−.148***	−.414	.242	−.073
R^2^	.652			.619		
F-value	34.743***			30.169***		
ΔR^2^	.199			.123		

*p < .05, **p < .01, ***p < .001

When negative burden of caring was analyzed, the apportioned and symbolic types revealed less impact than the remote type (β = −0.612, *p* < 0.001 & β = −0.271, p < 0.001). However, the result for the individualized type was not significant. Thus, it is possible to claim that grandparents of the individualized type did not feel a greater negative sense of burden of caring than did those of the remote type. Finally, the results showed that grandparents of the apportioned type felt a stronger impact of positive feelings such as satisfaction and happiness than of negative feelings such as fatigue and burden.

As can be seen in Table 5, with respect to involvement level, when time spent nurturing grandchildren increased**,** positive burden of caring decreased (*p* < .001). However, for negative burden of caring, when nurturing time increased, negative burden of caring increased (β = 0.210, *p* < 0.001). This means that when nurturing time increased, positive feelings such as happiness and satisfaction decreased, but negative feelings such as fatigue and constraint increased.

**Table 5:** Effect of involvement level on burden of caring

	Positive burden of caring			Negative burden of caring		
	B	SE B	β	B	SE B	β
Involvement level	−.033	.008	−.195***	.049	.011	.210***
R^2^	.486			.534		
F-value	20.884***			25.345***		
	.033			.038		

*p < .05, **p < .01, ***p < .001

Table 6 shows the mediating effect of each of the three non-baseline grandparenting role types on each of the three measures of psychological wellbeing. As can be seen, when the demographic variables (participants characteristics)—gender, age, spouse, religion, education, number of children, housemates, material support, health and living standard—were controlled, all three of the non-baseline grandparenting role types were found to have positively significant effects on meaning of life compared to the remote type. As with the results regarding positive burden of caring, the apportioned type showed the strongest effect (β = 0.765, *p* < 0.001). With respect to stress, all of the grandparenting role types were strongly negatively significant, with F value at 26.231% and the change of R^2^ was13.4%. Compared to the remote type, the apportioned type showed the strongest positive effect (β = 0.631, *p* < 0.001), followed by the symbolic type (β = 0.331, p < 0.001), whereas the individualized type showed a negative effect (β = −0.090, *p* < 0.005). In the case of depressive mood, the apportioned type had a positively significant relationship, meaning that it may have contributed to increased depressive mood when compared to the remote type. However, the symbolic and individualized types showed negatively significant relationships with depressive mood, which may translate into a positive impact (benefit) on this measure of psychological wellbeing as compared to the apportioned and remote role types.

**Table 6:** Mediating effect of grandparenting role type on measures of psychological wellbeing

	Meaning of life			Stress			Depressive mood		
	B	SE B	β	B	SE B	β	B	SE B	β
Apportioned type	1.494	.083	.765***	−.973	.112	.631***	.113	.026	.389***
Symbolic type	1.314	.117	.385***	−.891	.157	.331***	.116	.037	−.230**
Individualized type	.822	.147	.147***	−.397	.197	−.090*	.135	.046	−.163**
R^2^	.856			.586			.353		
F-value	110.405***			26.231***			10.093***		
ΔR^2^	.198			.134			.163		

*p < .05, **p < .01, ***p < .001

Table 7 shows the mediating effect of involvement level on each of the three measures of psychological wellbeing. Increased involvement appeared to show a significant positive correlation with meaning of life (*P* < .001). In terms of stress, when nurturing time increased, stress appeared to decrease, i.e., a negative correlation, which was found to be statistically significant. Thus, it is possible to claim that when the grandparents spent more time nurturing their grandchildren, they tended to experience more satisfaction, happiness and meaning in their lives than those who did not participate in nurturing their grandchildren. However, for depressive mood, when nurturing time increased, no change was observed.

**Table 7:** Mediating effect of involvement level on measures of psychological wellbeing

	Meaning of life			Stress			Depressive mood		
	B	SE B	β	B	SE B	β	B	SE B	β
Involvement level	.004	.009	.018	−.024	.009	−.134**	.003	.002	−.101
R^2^	.658			.467			.172		
F-value	42.562***			19.390***			4.575***		
ΔR^2^	.000			.015			.009		

*p < .05, **p < .01, ***p < .001

As seen in Table 8, after positive burden of caring was controlled, the apportioned, symbolic and individualized grandparenting role types were all found to be statistically significant (*P* < .001) in terms of their mediating effects on the dependent variable meaning of life. For the apportioned type, the value of the coefficient (β) decreased from 0.765 to 0.677. Thus, it is possible to claim that there was a partial mediating effect. In case of the symbolic role type, the value of the coefficient decreased from 0.385 to 0.341. In case of the individualized type, the value of the coefficient decreased from 0.147 to 0.130. These changes were statistically significant, and therefore it is possible to claim that there was a partial mediating effect for all three grandparenting role types compared to the remote type.

**Table 8:** Mediating effect of grandparenting role type on measures of psychological wellbeing after controlling for positive burden of caring (PBC)

	Meaning of life			Stress			Depressive mood		
	B	SE B	β	B	SEB	β	B	SEB	β
PBC	−.157	.056	−.114**	.450	.071	.414***	.033	.018	.164
Apportioned type	1.323	.102	.677***	−.484	.129	−.314***	.149	.033	.514***
Symbolic type	1.163	.127	.341***	−.461	.160	−.171**	−.084	.040	−.167*
Individualized type	.727	.149	.130***	−.126	.188	−.028	−.115	.047	−.138*
R^2^	.861			.646			.362		
F-value	105.992***			31.241***			9.721***		
ΔR^2^	.005			.060			.009		

*p < .05, **p < .01, ***p < .001

With respect to stress, the change in the coefficient value (β) was statistically significant for the apportioned and symbolic types. In the case of the apportioned type, the value decreased from 0.631 to −0.314, while for the symbolic type it decreased from 0.331 to −0.171. Meanwhile, for the individualized type the value decreased from −0.090 to −0.028, showing a partial mediating effect for stress with respect to positive burden of caring for this role type.

With respect to depressive mood, unlike meaning of life or stress, the coefficient value (β) increased for one of the role types, namely the apportioned type, for which the change from 0.389 to 0.514 showed no mediating effect. However, the coefficient value decreased from −0.230 to −0.167 for the symbolic type and from −0.163 to −0.138 for the individualized type. Thus, it showed mediating effects in both of these latter cases.

As seen in Table 9, the findings supported the hypothesis of negative burden of caring as a mediator for the apportioned, symbolic and individualized grandparenting role types. All mediating findings were statistically significant (*P* < 0.05) for meaning of life. The coefficient value (β) decreased from 0.765 to 0.712 for the apportioned type, from 0.385 to 0.362 for the symbolic type and from 0.147 to 0.140 for the individualized type. These results may show a partial mediation that is significant at the level of *p* < 0.001.

**Table 9:** Mediating effect of negative burden of caring (NBC) for grandparenting role type on measures of psychological wellbeing

	Meaning of life			Stress			Depressive mood		
	B	SE B	β	B	SE B	β	B	SE B	β
NBC	−.085	.039	−.085*	.180	.051	.229**	.009	.012	.059
Apportioned type	1.392	.095	.712***	−.757	.126	−.491***	.123	.030	.425***
Symbolic type	1.233	.122	.362***	−.718	.161	−.267***	−.108	.039	−.214**
Individualized type	.787	.147	.140***	−.323	.194	−.073	−.131	.047	−.158**
R^2^	.859			.606			.354		
F-value	104.433***			26.362***			9.387***		
ΔR^2^	.003			.020			.001		

*p < .05, **p < .01, ***p < .001

With respect to stress, after negative burden of caring was controlled, the change in the coefficient value (β) for each of the role types was as follows: apportioned type decreased from 0.631 to −0.491, symbolic type decreased from 0.331 to −0.267 and individualized type decreased from −0.090 to −0073. In this regard, the apportioned and symbolic types showed significant positive correlations, but the individualized type showed a very significant correlation. Thus, the impact of each of the role types showed a mediating effect of negative burden of caring.

With respect to depressive mood, after negative burden of caring was controlled, the coefficient value (β) for apportioned type increased from 0.389 to 0.425, showing no mediating effect. However, the value for the symbolic type decreased from −0.230 to −0.214 and the value for the individualized type decreased from −0.163 to −0.158. Therefore, it is possible to claim that there was a partial mediating effect for both the symbolic and the individualized types.

As can be seen in Table 10, after positive burden of caring was controlled, involvement level correlated positively with meaning of life, and the coefficient value (β), −0.071, was significant at the level *P* < 0.05. However, the coefficient value increased from 0.018, so the results showed a suppression effect rather than the hypothesized mediating effect.

**Table 10:** Impact of involvement level on psychological wellbeing after controlling for positive burden of caring (PBC)

	Meaning of life			Stress			Depressive mood		
	B	SE B	β	B	SE B	β	B	SE B	β
PBC	−.630	.060	−.458***	.600	.008	.553***	−.011	.017	−.055
Involvement level	−.016	.008	−.071*	−.005	.008	−.026	−.004	.002	−.112
R^2^	.766			.625			.173		
F-value	66.030***			33.562***			4.223***		
ΔR^2^	.108			.157			.001		

*p < .05, **p < .01, ***p < .001

With respect to stress, after positive burden of caring was controlled, the coefficient value (β) for involvement level decreased from −0.134 to −0.026. In this regard, it is possible to claim that positive burden of caring had a mediating effect. When positive burden of caring increased, stress also increased; however, when involvement level increased, stress decreased slightly, indicating a mediating effect of positive burden of caring.

For depressive mood, the change in the coefficient value (β) was not significant even after positive burden of caring was controlled. Nonetheless, since the coefficient value increased slightly, from −.101 to −.112, it is possible to claim that there was no mediating effect.

Table 11 shows the results of the examination of the mediating effect of negative burden of caring on the impact of involvement level on the three variables of psychological wellbeing. For meaning of life, the coefficient value (β) increased from 0.018 to 0.104. Thus, it is possible to claim that there was no mediating effect with regard to this variable. With respect to stress, the coefficient value increased from −0.134 to −0.241, so it was also possible to claim that there was no mediating effect. For depressive mood, the coefficient value decreased slightly, from −0.101 to −0.087. However, even before negative burden of caring was controlled, the change was not significant. Thus, it is not possible to claim that there was no mediating effect.

**Table 11:** Impact of involvement level on psychological wellbeing after controlling for negative burden of caring (NBC)

	Meaning of life			Stress			Depressive mood		
	B	SE B	β	B	SE B	β	B	SE B	β
NBC	−.406	.048	−.409***	.402	.047	.513***	−.009	.013	−.064
Involvement level	.024	.009	.104**	−.044	.008	−.241***	−.003	.002	−.087
R^2^	.736			.570					
F-value	56.279***			29.045***					
ΔR^2^	.078			.123					

*p < .05, **p < .01, ***p < .001

Table 12 shows the results of the Sobel test that was conducted to verify the mediating effect of positive and negative burden of caring on the impact of involvement level on each of the three measures of psychological wellbeing. It also summarizes the findings regarding the mediating effects of positive and negative burden of caring on the impact of role type and involvement level on the variables associated with psychological wellbeing in the present study.

**Table 12:** Results of sobel test

	Meaning of life	Stress	Depressive mood
Positive burden of caring			
Apportioned type	2.725**	−5.559***	−1.879
Symbolic type	2.612**	−4.754***	−1.841
Individualized type	2.22*	−3.141**	−1.685
Involvement level	3.839***	−4.119***	0.825
Negative burden of caring			
Apportioned type	2.116*	−3.276**	−.357
Symbolic type	1.998*	−2.883**	−.356
Individualized type	1.346	−1.539	−.350
Involvement level	−3.941***	3.951***	−1.159

*p < .05, **p < .01, ***p < .001

All in all, as shown in Table 13, both positive and negative burden of caring had partial mediating effects on the impact of role type on meaning of life and stress for all three role types, except that there was no mediating effect for negative burden on the impact of individualized role type on stress, while depressive mood showed no mediating effects. In addition, with respect to involvement level, the only positive finding was a full mediating effect of positive burden of caring on the impact on stress.

**Table 13:** Summary of mediating effects of positive and negative burden of caring (PBC and NBC) on the impact of role type and involvement level on psychological wellbeing

	Meaning of life		Stress		Depressive mood	
	PBC	NBC	PBC	NBC	PBC	NBC
Apportioned type	Partial	Partial	Partial	Partial	–	–
Symbolic type	Partial	Partial	Partial	Partial	–	–
Individualized type	Partial	Partial	Partial	–	–	–
Involvement level	–	–	Full	–	–	–

### Interaction effects

To examine the interaction effect of the feeling of respect, a hierarchical regression was used. Only significant and meaningful interactions are reported here.

As seen in Table 14, when grandparents felt respected, stress decreased. Stress increased with positive burden of caring, but the interaction was not statistically significant (β = 0.051, significance level *p* < 0.5).

**Table 14:** Interaction effect between feeling of respect and positive burden of caring (PBC) for stress

	Stress			Stress			Stress		
	B	SE B	β	B	SE B	β	B	SE B	β
Apportioned type	−.522	.143	−.338***	−.294	.144	−.191*	−.248	.158	−.161
Symbolic type	−.373	.185	−.139*	−.221	.180	−.082	−.165	.196	−.061
Individualized type	−.229	.192	−.052	−.071	.186	−.016	−.053	.188	−.012
Respect	−.340	.071	−.393***	−.206	.073	−.239**	−.208	.073	−.241**
PBC				.372	.075	.343***	.433	.114	.399***
R * PBC							.068	.095	.051
R^2^	.622			.657			.658		
F-value	28.180***			30.535***			28.600***		
ΔR^2^	.036			.035			.001		

*p < .05, **p < .01, ***p < .001

As seen in Table 15, the interaction between feeling of respect and negative burden of caring showed a significant negative correlation for meaning of life (β = −0.065, *p* < 0.05). With respect to stress and depressive mood (not shown), the interaction effects were not statistically significant (stress: β = −0.019, p < 0.05; depressive mood: β = −0.043, p < 0.05). However, compared to the results for the interaction effect between respect and positive burden of caring, these results support the view that participants in the three non-baseline role types who felt respected *tended to experience more positive burden of caring* in the form of feelings of satisfaction than negative burden of caring in the form of stress and depressive mood.

**Table 15:** Interaction effect between feeling of respect and negative burden of caring (NBC) for meaning of life

	Meaning of life			Meaning of life			Meaning of life		
	B	SE B	β	B	SE B	β	B	SE B	β
Apportioned type	1.005	.101	.515***	.977	.105	.500***	.904	.111	.462***
Symbolic type	.754	.131	.212***	.736	.132	.216***	.636	.140	.187***
Individualized type	.640	.136	.114***	.630	.136	.113***	.670	.137	.120***
Respect	.368	.050	.336***	.358	.051	.327***	.366	.051	.334***
NBC				−.034	.036	−.034	−.094	.047	−.095*
R * NBC							−.093	.045	−.065*
R^2^	.882			.883			.885		
F-value	128.587***			120.006***			114.248***		
ΔR^2^	.026			.000			.002		

*p < .05, **p < .01, ***p < .001

With respect to the impact of involvement level on psychological wellbeing (Table 16), feeling of respect showed an interaction effect on the mediating effect of negative burden of caring only with regard to meaning of life (β = −0.117 at *P* < 0.01).

**Table 16:** Interaction effect of feeling of respect on the mediating effect of negative burden of caring (NBC) on the effect of involvement level on meaning of life

	Meaning of life			Meaning of life			Meaning of life		
	B	SE B	β	B	SE B	β	B	SE B	β
Involvement level	−.009	.007	−.040	−.001	.007	−.006	−.003	.007	−.015
Respect	.696	.044	.635***	.618	.051	.564***	.594	.050	.542***
NBC				−.132	.044	−.133**	−.224	.051	−.226***
R* NBC							−.167	.048	−.117**
R^2^	.831	.837	.845						
F-Value	98.925***			95.020***			93.173***		
ΔR^2^	.172			.006			.008		

*p < .05, **p < .01, ***p < .001

## Discussion and Practice Implications

The study sought to examine how role type (and perceptions of role type), involvement level, burden of care, and respect impacted grandparents’ psychological well-being (see Fig. 1). Overall, in terms of the study’s first two hypotheses, the analyses demonstrated that grandparent role type significantly predicted psychological well-being. Specifically, participants in the non-baseline role types (i.e., those who participated in grandparenting) tended to experience a high sense of life meaning and reported relatively low stress and depressive symptoms. Thus, participation in grandparenting was related to increased levels of positive affect (such as happiness, satisfaction, and joy) and low levels of negative affect (such as stress, frustration, depression, and fatigue). With respect to level of involvement, stress tended to decrease (β = −0.134) when involvement was high, but no relationship emerged with feelings of meaning or depressive mood.

Moreover, grandparenting role type significantly predicted burden of care, in which both a positive and negative sense of burden was low for all three non-baseline role types compared to the remote type. In other words, participation in grandparenting was related to a mixture of both positive and negative affect. With respect to involvement level, the amount of time participants devoted to grandparenting was related to a decrease in positive affect and increased negative affect.

In addition, as predicted in hypothesis 3, burden of care mediated the relationship between role type and involvement level on psychological well-being. When both positive and negative care burden were included as mediators, reported life meaning increased, as well as above-baseline stress levels; however, there was no association with depressive mood. Involvement level had a mediating effect for positive care burden predicting increased stress.

Finally, as predicted in hypothesis 4, respect moderated the mediating effect of care burden on role type and involvement level in predicting psychological well-being. Specifically, when grandparents across the other three role types felt respected by their adult children, they reported a greater sense of life meaning and exhibited less stress and depressive symptoms when compared to the baseline remote type grandparents or those grandparents who did not feel respected.

### Implications for Practice

At least four major implications for social work policy and practice can be drawn from these results. First, since it appears that grandparents’ participation in nurturing their grandchildren can influence their psychological well-being, social work policy makers should strive to provide resources and support for seniors in order for those who might benefit to engage more readily with these important roles. In South Korea, where the study was conducted, this could be an important area for raising public and institutional awareness in light of the statistical increase in suicides among economically challenged elderly individuals noted at the beginning of this paper (Seoul Statistical Office 2005).

Second, given that significant moderated mediation emerged in terms of care burden and grandparent roles and involvement, social work practitioners should be aware of the important interactions among grandparents, adult children, and grandchildren when providing family counselling and other resources. Furthermore, efforts should be made to promote and achieve synergy across generations within the family unit. Such approaches can have benefits for all family members, including helping grandparents feel an increased sense of pride, rather than a sense of isolation, while providing young parents with models for positive family value systems. Intergenerational interactions could also help younger individuals with regard to negative perceptions they might harbour toward ageing, enabling them to develop a more realistic attitude toward the ageing process.

Third, as compared to social exchange theory (Marbach 1995) or some other exchange model, the present study suggests that there are advantages to applying dynamic practice models such as Bowen’s (1971) family system therapy to social work practice. Such an approach is especially appropriate in a South Korean context, where most families encompass more than two generations. According to Bowen, individuals are best understood through an assessment of the entire family system and each individual’s role. The goal of family system therapy is to help family members improve communication, solve family problems, understand and handle special family situations, and create a more functioning home environment. In this approach, problems are treated by changing the way the system works rather than by trying to ‘fix’ a specific member. Thus, family system therapy is used to help both families and individual family members of all ages cope with and overcome various challenges, including quests toward emotional fulfilment within new roles across the age spectrum.

Finally, the results have implications regarding the impact of grandparent attitudes and behaviour patterns within the changing social dynamics of the twenty-first century. Family patterns in South Korea are changing, with significant demographic shifts that include rapid growth in multi-generational families, later marriages, fewer children, and increased family breakdown and re-formations. Furthermore, these shifts are occurring over a much longer lifetime than was once the norm. Moreover, while the relative number of children is decreasing, with both the elderly population and percentage of working mothers increasing in South Korea, many grandparents are taking on new, active caregiving roles. Thus, it is vital that social work practitioners be prepared to assist elderly clients and their families in order to address these evolving roles and the impacts they have on the family unit.

### Limitations and Research Implications

Several limitations of the present study should be noted. First, the sample consisted of only 255 grandparents recruited from 14 kindergartens in Seoul. Although some of the sample characteristics appeared to match the broader South Korean population, the sample size and geographic distribution were too limited for generalizability.

Second, the ages of both the grandparents (participants) and their grandchildren were truncated. Grandparents who participated were mostly in the 61–65 age range, while their respective grandchildren were all between 3 and 5 years old. Thus, the study sample was also limited in scope regarding representation of the possible grandparent-grandchild age spectrum.

Third, women vastly outnumbered men in the current sample (224 grandmothers and only 31 grandfathers). Thus, future research should attempt to oversample from grandfathers in order to provide more valid gender comparisons.

Fourth, some participants did not complete all of the questionnaire measures (or, in some cases, individual items within these measures) thoroughly or thoughtfully. An attempt was made to prevent this shortcoming by providing participants with certain necessities in connection with their participation; however, this did not fully eliminate the need to exclude some of the data.

Finally, as the study used a cross-sectional design, the results cannot contribute substantially to the understanding of how the parameters considered here might change over time. Thus, future studies should address progress in participants’ grandparenting experiences, including possible changes in the impact of moderating and mediating factors.

## Conclusion

The present study contributed to the understanding of factors impacting grandparents’ psychological well-being when fulfilling their grandparenting roles. Overall, the results suggest that although Grandparenting can be stressful, it can also be a positive experience that adds new vitality to the lives of elderly adults. As a society, it is important that we provide grandparents with understanding, support, and respect as they carry out their roles. To contribute to this effort, further research in this area should be encouraged, and legislators, policy makers, and practitioners should endeavour to implement research-based recommendations in ways that promote elder adult health and well-being.

## Footnotes

1 Y1 = Burden of caring; Y2 = Psychological wellbeing; X1 = Grandparenting role type; X2 = Involvement Level; Z = Respect.
